# Do Antimicrobial Proteins Contribute to Overcoming the Hidden Antifungal Crisis at the Dawn of a Post-Antibiotic Era?

**DOI:** 10.3390/microorganisms7010016

**Published:** 2019-01-11

**Authors:** László Galgóczy, Florentine Marx

**Affiliations:** 1 Institute of Plant Biology, Biological Research Centre, Hungarian Academy of Sciences, Temesvári krt. 62, H-6726 Szeged, Hungary; galgoczi.laszlo@brc.mta.hu; 2 Department of Microbiology, Faculty of Science and Informatics, University of Szeged, Közép fasor 52, H-6726 Szeged, Hungary; 3 Biocenter, Division of Molecular Biology, Medical University of Innsbruck, Innrain 80-82, A-6020 Innsbruck, Austria

The incidence of fungal infections has been grossly underestimated in the past decades as a consequence of poor identification techniques and a lack of regular epidemiologic surveys in low- and middle-income countries. The most affected areas of the world are the countries where some routinely administrated and effective antifungal drugs are not available, especially for people under living standards [1]. Approximately one quarter of the worldwide population suffers from superficial mycoses [2], which is the fourth most common illness on Earth after headaches and dental caries [3]. In the immunocompetent patient, cutaneous fungal infections are non-life-threatening, but associated with social embarrassment and stigma [1], because they might be recurrent and difficult to eradicate [4]. Patients who suffer from impaired and weak immune status due to infections (e.g. HIV), hematological malignancies, who receive immunosuppressive therapy or necessitate intensive care (e.g. organ transplantation) are at high risk for microbial infections in general and more specifically to develop severe invasive mycoses, such as aspergillosis, candidiasis, cryptococcosis, histoplasmosis, talaromycosis and pneumocystosis with fatal outcome [5,6]. It has been estimated that the number of people that die from fungal diseases per year is > 1.5 million and outreaches the numbers that die from tuberculosis or malaria [2].

Plant pathogenic fungi also pose severe risk to human and animal health, and food supply. Crops, worth billions of € per year, are destroyed by plant pathogenic fungi that constitute the main group of phytopathogens. Mycotoxin-contaminated crops, vegetables, fruits, and animal feeds are continuously increasing in Europe in the last years [7]. Moreover, fungal bio-deterioration of buildings, paintings, object arts and books also have impact on our cultural heritage [8]; furthermore, indoor molds affect human health with impairing the immune status of healthy individuals [9]. Finally, the emergence of novel fungal diseases in plants and animals, as a result of climate change, is affecting global sustainability and biodiversity [10,11].

The number of effective antifungal drugs to prevent fungal contaminations and treat life-threatening mycoses is modest in comparison to the armamentarium available for antibacterial treatment strategies [12]. The high similarity between fungal pathogen and host in terms of cellular morphology, physiology and metabolism hampers the identification and development of new compounds that interfere with targets that are unique to fungi and guarantee improved tolerance or at least minimize severe side effects in the host. Consequently, pesticides and feed supplements in agriculture and animal breeding to prevent or reduce fungal infections belong to the same class of active agents that are also applied in human medical treatment. Since the early 1990’s, when triazoles were introduced in antifungal therapy, a pronounced increase in *Candida* and *Aspergillus* species was observed that were less susceptible or even resistant to these licensed drugs [13,14]. In contrast to fungi that are intrinsically resistant against certain drug classes (e.g. non-*albicans Candida* species, NAC) or are multidrug-resistant (e.g. *Candida glabrata* [15], *Candida auris* [16], *Fusarium solani* [17], and *Lomentospora* (formerly *Scedosporium*) *prolificans* [18]), a resistance mechanism can also be acquired following repeated and long-term exposure to antifungal drugs. This accounts for iatrogenic azole resistance development in fungi but also the emergence of azole-resistant aspergillosis as a consequence of wide agricultural use of this class of antifungal drugs [19]. In the latter case azole therapy may fail in the host who has never been treated with azoles before, but suffers from infection with an azole-resistant *Aspergillus fumigatus* environmental strain [20].

Thus, early and accurate diagnosis is essential to promote the proper administration of the most effective antifungal therapy and reduce morbidity and mortality. In parallel, effective surveys need to be implemented that ensure the use of fungicides in food production and animal and human medicine. Furthermore, collaborative efforts are required to identify and develop new compounds with high fungal specificity and novel antifungal mechanisms to overcome the limitations of resistance development and adverse toxicity in the host.

In this respect, nature constitute a rich source for proteins and peptides that promise future drug development. Small, cysteine-rich, cationic proteins that are produced by the most diverse organisms throughout the phylogenetic tree are potential candidates for new antifungal medicines and therapeutic strategies [21]. Most promising thereby are antimicrobial proteins and peptides (AMPs) originating from filamentous fungi belonging to the class Eurotiomycetes (division Ascomycota). Numerous representatives have been extensively analyzed to date, reviewed in [22,23,24,25] and even more await their identification and characterization as the genomes of filamentous ascomycetes harbor information for still unidentified proteins with antimicrobial potential [26,27,28,29]. Deep mining of fungal genome databases and accurate phylogenetic analyses promote these efforts [29]. One obstacle in protein isolation might be the unknown gene regulation and/or very low protein expression levels. This, however, can be overcome by using (heterologous) expression systems that allow the generation and purification of recombinant proteins in sufficient quantity and of high quality that ensure thorough characterization of their structure and function [27,30]. Also, chemical synthesis has been developed for cysteine-rich AMPs, coping with the challenge of correct disulfide bonding within the protein during the synthesis process [31,32].

AMPs from Eurotiomycetes share common features: (i) they are encoded as pre-pro proteins with a signal- and a pro-sequence, which both are cleaved during protein processing and secretion into the culture broth; (ii) the mature proteins consist of ca. 50–60 amino acids (Mw: ~6000 Da), lack any posttranslational modifications, and three to four disulfide bridges stabilize a compact β-fold structure; (iii) all proteins investigated in detail so far exhibit high stability against harsh environmental conditions and (iv) exclusively inhibit the growth of human- and plant-pathogenic fungi in a fungistatic and/or fungicidal manner at µM concentrations [21]. In addition, antiviral activity has been reported in one case [33]. However, some differences exist in species specificity, antifungal efficacy, and mode of action, which are distinct from that of conventional antifungal drugs [21].

Their antifungal mechanisms are intensively studied nowadays. On the one hand, the use of genetically manipulated fungal strains allowed to define pathways involved in protein toxicity [34,35,36,37,38]. On the other hand, comprehensive nuclear magnetic resonance analyses provided detailed knowledge on the solution structure of AMPs and proposed protein motifs that might play an important role in protein function and host interaction [32,33,39,40]. Unfortunately, no fungal-specific target molecule for AMPs has been identified so far, which restrains the development of new, safely applicable AMP-based antifungal strategies. However, the availability of accurate AMP solution structures deposited in the protein data bases (such as the worldwide Protein Data Bank [41]) provide a valuable source for *in silico* homology modelling and molecular dynamics simulations to propose not only the structure of uncharacterized AMP candidates but also the mechanistic way of action [42]. These structural and functional predictions pave the way towards the modification of natural proteins and peptides applying rational design tools or combinatorial approaches to generate novel AMPs with improved efficacy against plant and human pathogenic fungi [29,32,43,44]. As the chemical synthesis of peptides and proteins becomes more cost-effective [45], AMPs and short peptide derivatives have significant commercial potential on the global market to be produced and applied as new antifungal compounds in the future [46].

In vitro and in vivo experiments approved fungal AMP tolerance of mammalian and plant cells and efficacy in reducing fungal pathogen load in plant, fruit, and animal models, corroborating their suitability for the development of new antifungal strategies in medical treatment, plant protection and food preservation [25,29,33,47,48,49,50,51,52,53,54,55,56,57,58,59,60,61,62,63,64,65].

Apart from the AMPs originating from fungi of the class Eurotiomycetes, small antimicrobial peptides from various mitosporic filamentous fungi belonging to the genus *Trichoderma* (class Sordariomycetes) have been characterized that form a unique class of so-called peptaibols. These small peptides (ca. 5–20 amino acid long; Mw: ~500–2200 Da) contain unusual amino acids and are synthesized by non-ribosomal peptide synthases. Their antibacterial and antifungal activity is closely linked with a helical structure, that favors aggregation and ion channel formation in lipid bilayers causing membrane damage [66,67,68,69].

In this Special Issue on "Antimicrobial Proteins in Filamentous Fungi", the contributors addressed achievements in this research field and posed questions that need to be answered to better understand the nature of AMPs and to push forward the development of novel AMP-based antimicrobial strategies and to overcome antimicrobial resistance.

Heredero et al. aimed in their work at the rational design of chimeric protein:peptide molecules with improved antifungal efficacy. The combination of the *Penicillium digitatum* AMP AfpB with the hexapeptide PAF26 resulted in biotechnologically produced protein chimeras that allowed new insights into AMP design, structure, and function [70].

The paper of Marik et al. focuses on the characterization of new peptaibols from *Trichoderma gamsii* and *Trichoderma koningiopsis*, belonging to clade Viride, showing variations in antimicrobial activity that can be assigned to differences in the cell wall structure of the target organisms [71].

The study by Delgado et al. proved the potential of a *Penicillium chrysogenum* AMP, the PgAFP in food protection. PgAFP combined with the food ripening-yeast *Debaryomyces hansenii* effectively reduced *Aspergillus parasiticus* growth and aflatoxin contamination in dry-fermented sausage and cheese [72].

Unravelling the AMP structure and function provides important information for the improvement of AMP efficacy and the development of new treatment strategies by rational drug design. An overview on structure peculiarities of β-strand disulfide AMPs from ascomycetous origin and possibilities of their production by modern synthetic chemistry methods and recombinant technology is given in the mini-review by Váradi et al. [73].

Finally, the review of Meyer and Jung [74] compares structure and function of AMPs from Ascomycetes with those of bacterial cannibal toxins and redirects our narrow human conception of AMPs as purely bioactive molecules towards their important role in controlling different cellular processes to ensure optimal fitness of the producing fungal organism.

In conclusion, we want to thank all contributors to this Special Issue on AMPs in filamentous fungi for their participation. The papers published in this Special Issue provide a most valuable overview on the functional and structural complexity of the members of this AMP group and reflects the big efforts made by this community to unravel the most divers aspects in understanding fungal AMPs. This should encourage the worldwide scientific community engaged in AMP science to specifically expand their attention towards AMPs from filamentous fungi, many of which still await identification and characterization.
